# The Prevalence of Infection and Sepsis Associated Dysphagia in Hospitalised Patients: A Retrospective Cross-sectional Study

**DOI:** 10.1007/s00455-026-10931-w

**Published:** 2026-03-04

**Authors:** Ayodele Sasegbon, Karim Bastawisy, Ivy Cheng, Shaheen Hamdy

**Affiliations:** 1 Division of Diabetes, Endocrinology and Gastroenterology, School of Medical Sciences, Centre for Gastrointestinal Sciences, Faculty of Biology, Medicine and Health, Salford Royal Foundation Trust, University of Manchester, Manchester, UK; 2 Royal Albert Edward Infirmary, Wigan, UK; 3 Academic Unit of Human Communication, Learning, and Development, Faculty of Education, The University of Hong Kong, Hong Kong, China

**Keywords:** Dysphagia, Hospital, Infection, Sepsis

## Abstract

Little is known about the relationships between infections, sepsis and dysphagia. In this study, we aimed to assess the prevalence and factors associated with dysphagia in patients admitted to hospital with infections or sepsis. Participants ≥ 18 years admitted to hospital with infections or sepsis caused by pneumonia, urine tract infections or cholecystitis were recruited from an NHS Trust in the UK. Electronic patient record and clinical coding staff screened patient data from 01/02/2022 to 01/02/2023. Patients with previous dysphagia, acute head injuries or stroke were excluded. Those that remained had data extracted from their notes, including age, gender, length of stay (LOS), co-morbidities, and mortality. Data was then analysed using statistical tests, including Logistic regression. Over the study period, 4475 patients (M:2031, F:2444) were admitted with infections (Pneumonia 2465, UTI 1888 and Cholecystitis 401). Of these, 189 (4.2%) developed dysphagia during their inpatient stay (Pneumonia 6.0%, UTI 3.5%, Cholecystitis 0.8%). Additionally, 865 patients (M:434, F:431) were admitted with sepsis (Pneumonia 535, UTI 413 and Cholecystitis 55). Of these, 54 (6.2%) developed dysphagia (Pneumonia 9.3%, UTI 3.4%, Cholecystitis 3.6%). Logistic regression revealed that increasing age, the presence of sepsis, delirium, dementia and Parkinson’s disease (PD) were significantly associated with an increased risk of dysphagia (OR = 1.02, 1.74, 1.60, 2.43, 3.88; *P* = 0.002, 0.013, 0.008, < 0.001, < 0.001). Our study has identified that dysphagia is associated with infections and sepsis. Additionally, patients with PD, delirium or dementia who are not known to have swallowing impairments are at risk of developing dysphagia should they be admitted with infections or sepsis.

**Supplementary Information:**

The online version contains supplementary material available at 10.1007/s00455-026-10931-w.

## Introduction

Dysphagia is defined as difficult or disordered swallowing [1]. Unfortunately, information regarding its epidemiology is patchy, as dysphagia is not a distinct disease, but rather an aftereffect of many disease processes [2]. The physiological process by which swallowing occurs is complex, involving the coordinated contraction of numerous muscles in the head and neck, orchestrated by the brainstem with input from higher centres within the brain [1]. Dysphagia can occur following the disruption of any part of this complex physiological process by numerous diseases [1]. When dysphagia is present, it can cause numerous deleterious effects, such as dehydration, malnutrition, aspiration, increased hospital length of stay, and increased mortality [3]. Due to the well-known negative effects of dysphagia and subsequent aspiration in patients following strokes, most epidemiological work has been performed in this area. These studies have established that oropharyngeal dysphagia is very common following strokes, with an incidence ranging from 29% to > 80% [4]. Dysphagia is also commonly found in the general population, affecting 16% of people [5].

Other than those with neurological diseases, studies have traditionally highlighted two groups of patients at particular risk of dysphagia. These are the elderly and the critically ill. In elderly individuals, the process of swallowing shows evidence of reduced efficiency, which increases their risk of decompensation and subsequent dysphagia [6]. Multiple studies have also shown that critically ill patients are at an increased risk of dysphagia. This is thought to be due, in part, to critical illness associated neuromuscular changes and critical illness polyneuropathy (CIP) [7, 8]. More recently, a small number of studies have also identified sepsis as being associated with dysphagia. While infections can be defined as the growth of pathogens within the body, which prompt an immune response [9], the current definition of sepsis has moved away from the older systemic inflammatory response syndrome (SIRS) criteria and is now defined as “life‑threatening organ dysfunction caused by a dysregulated host response to infection” [10].

In 2014, Zielske et al. prospectively studied 30 patients with severe sepsis in an intensive care unit (ICU) and found that 63% of patients with severe sepsis had dysphagia compared to 23% of patients without sepsis [8]. In addition to its prospective design, the strengths of this study included the fact that dysphagia was objectively assessed using fibreoptic endoscopic evaluation of swallowing (FEES), the inclusion of multiple types of sepsis, including pneumonia, intrabdominal sepsis, and urosepsis, and the reassessment of patients following discharge. Following this, a similar small ICU and high dependency unit (HDU) based study was performed in 2023 by Hongo et al. [11]. In this study, of 101 patients, the authors showed that 57% of patients with severe sepsis and shock had dysphagia. The strengths of this study were its larger number of patients and incorporation of multiple types of sepsis. Between the two ICU studies, in 2018, Sasegbon et al. showed that sepsis was associated with oropharyngeal dysphagia in older patients on medical wards who were not critically unwell [12]. In this study, the prevalence of sepsis-associated dysphagia was found to be 16% of patients referred to speech and language therapists. Furthermore, they showed, for the first time, that in 10% of patients with sepsis-associated dysphagia, occurred without any other known risk factor for dysphagia being present, implying that sepsis itself was a potential cause of dysphagia [12]. Regarding recovery, the Hongo et al. and Sasegbon et al. studies found that 60% and 84% of patients, respectively, with sepsis-associated dysphagia did not return to their pre-septic swallowing baseline prior to discharge [11, 12]. Despite this, no patient in the Sasegbon et al. study had undergone speech and language therapy led swallowing rehabilitation exercises, which have been shown to improve swallowing function [12]. This implied that prompt swallowing rehabilitation may have led to improvements in swallowing function, dysphagia-associated complications, and quality of life. Across the three studies, two explanations for the occurrence of dysphagia have been proposed. The first by Zielske et al., suggested that dysphagia occurred in critically unwell patients with severe sepsis due to severe sepsis being linked to CIP [8]. While the second by Sasegbon et al., postulated that sepsis causes wasting of oropharyngeal muscles due to catabolism in a similar fashion to sepsis associated skeletal muscle wasting [12]. The resultant impairment would cause presbyphagic patients, or those with impaired swallowing to decompensate and develop frank dysphagia. To date, while there is some evidence supporting sepsis causing dysphagia via CIP [8], the oropharyngeal wasting effect proposed by Sasegbon et al. remains plausible but hypothetical.

Though these studies have notable strengths and have been useful in drawing attention to sepsis-associated dysphagia, they have several limitations. Those which apply to all three studies are that they are single site studies with relatively small numbers of patients. In more detail, the study by Zielske et al., was limited by its lack of randomisation, putting its findings at risk of selection bias, and the fact that no baseline assessment of swallowing was performed at the point of admission, meaning the temporal relationship between the occurrence of sepsis and subsequent dysphagia could not be conclusively established. The study by Hongo et al. was limited by its retrospective design, meaning that causation could not be established and the fact that no follow up took place following discharge. Lastly, the study by Sasegbon et al., was limited by its retrospective nature, the fact that it was restricted to elderly patients, and the fact that no post discharge follow-up occurred.

Despite what is known, there remain multiple gaps in our understanding of the relationship between sepsis and dysphagia. This is particularly the case in non-critically unwell patients. Most hospitalised patients with infections and sepsis are not critically unwell, which limits the generalisability of studies performed in critical care settings, where other factors like CIP and recent extubation may play a role. At present, the prevalence and characteristics of sepsis-associated dysphagia in patients admitted to hospital is not fully understood. Previous studies have focused on small numbers of patients in settings that are not entirely reflective of the general pool of hospital inpatients. Furthermore, no study has attempted to establish the existence of infection-associated rather than sepsis-associated dysphagia. In this study, we aim to quantify the prevalence of both infection and sepsis-associated oropharyngeal dysphagia in patients without preexisting dysphagia.

## Methodology

### Study Design and Participants

This study was designed as a retrospective cross-sectional study. Participants ≥ 18 years of age were selected from the electronic patient records (EPRs) of a large hospital trust in England. Electronic patient record administrators, clinical coding staff and members of the research team screened EPRs from 01/02/2022 to 01/02/2023 using the study inclusion and exclusion criteria. The study was granted ethical approval by the University of Manchester Proportionate University Research Ethics Committee (Ref:2024–21059-36783).

### Inclusion and Exclusion Criteria

All patients aged 18 or above who were admitted to hospital with infections or sepsis due to pneumonia, urine tract infections (UTI) or cholecystitis were included. The decision to study three types of infections/sepsis was made to establish the prevalence of dysphagia in commonly encountered infections affecting different organ systems, while reflecting previous published literature [12] and being feasible for the research team to complete.

Exclusion criteria were patients below 18 years; without infections or sepsis; those with infections or sepsis that were not due to pneumonia, UTIs or cholecystitis; those with previous dysphagia; and patients with acute strokes or head injuries.

## Data

### Definitions

In this study, information about infections, sepsis, oropharyngeal dysphagia, comorbidities, ICU admissions and mortality was predominantly obtained from the International Statistical Classification of Diseases: Tenth Revision (ICD-10) clinical codes in patients’ EPRs. This approach has been used in other published studies [13, 14] and allowed the relatively rapid screening and categorisation of large numbers of patient admissions during the study period. Furthermore, it allowed the easy identification and removal of patients who possessed the study exclusion criteria. As has been described in previous studies, the study team were aware that clinical coding data used in this manner is not perfectly accurate and inaccurate coding may result in the loss of some pertinent information [15]. For example, this study aimed to assess oropharyngeal dysphagia rather than oesophageal dysphagia. Yet despite the fact that they are coded differently, there is a risk that some oesophageal dysphagia was miscoded as oropharyngeal dysphagia or vice versa. This has the potential to increase the type 2 error rate of the study [16] and must be borne in mind when interpreting our findings.

### Dysphagia

Dysphagia is diagnosed within the hospital trust following a process by which clinical staff on wards suspect swallowing dysfunction to be present and refer patients for urgent further assessment by speech and language therapists (deglutologists). A diagnosis is then made following clinical or instrumental assessments.

### Preparation

All data was subject to a multistage process of preparation by clinical coding, EPR and research team members, to ensure maximal accuracy. Initially, all patients admitted with infections or sepsis during the study period were screened to remove any who bore any of the study exclusion criteria. Subsequently, all fully duplicated entries were excluded. Following this, patients with multiple similar but non fully duplicated entries from their inpatient stay had their records merged as appropriate. This pertained to patients who were moved to several wards during their hospital admission and, as a result, had several coded entries on the hospital database. Following this extensive process of preparation, the data was analysed.

### Key Data Extraction

Once screening and data preparation were completed, the following data were obtained from patients’ EPRs.

-  Gender
-  Age
-  Date of admission
-  Date of discharge (or date of death if died during admission)
-  Length of inpatient stay (LOS)
-  Type of infection (pneumonia, urine tract infection or cholecystitis)
-  Whether a diagnosis of sepsis was made
-  Whether dysphagia occurred during admission
-  Common comorbidities and those of particular clinical interest (Obesity, Chronic kidney disease (CKD), heart failure (HF), hepatic cirrhosis, chronic obstructive pulmonary disease (COPD), dementia (all causes), delirium (all causes), Parkinson’s Disease (PD), and motor neurone disease (MND))
-  Frailty
-  Whether patients were admitted to the ICU
-  In hospital mortality (all cause)

Records with missing data were excluded from further analysis.

It was recognised that the inclusion of some comorbidities which are known causes of dysphagia, such as PD, dementia and delirium, may introduce confounding. However, the decision was made to include these comorbidities to accurately determine the prevalence of sepsis-associated dysphagia as it occurs in routine clinical practice. This approach ensures the findings are clinically relevant and provides a more useful reference for clinicians working in emergency and acute medical settings. Furthermore, studies suggest that including similar confounding factors in regression analyses reduces bias and enables the assessment of the independent association between each factor and, in our case, dysphagia [17]. Lastly, the exclusion of any patient with previous dysphagia from the study was intended to reduce confounding as much as possible within the constraints of the study design.

### Statistical Analysis

Data were expressed as proportions and percentages where applicable. Comparisons were made between patients with and without dysphagia for total infections and sepsis, as well as for each type of infection or sepsis. Chi-squared (X^2^) and T-Tests were applied for group comparisons. Logistic regression was used to calculate the factors associated with dysphagia in patients presenting to hospital with infections. Due to the risk of multicollinearity in a study of this nature, appropriate diagnostic checks were performed. Additionally, with the number of variables being assessed, overfitting was avoided by ensuring an events per variable (EPV) of ≥ 10 [18]. The area under the receiver operating characteristic (ROC) curve (AUC) was used to assess the performance of the logistic regression model. Cox regression was used to assess the factors associated with increased LOS and mortality. Significance was set at *p* < 0.05. JASP (Version 0.19.3) software was utilised for all statistical analyses.

## Results

**Fig. 1:**
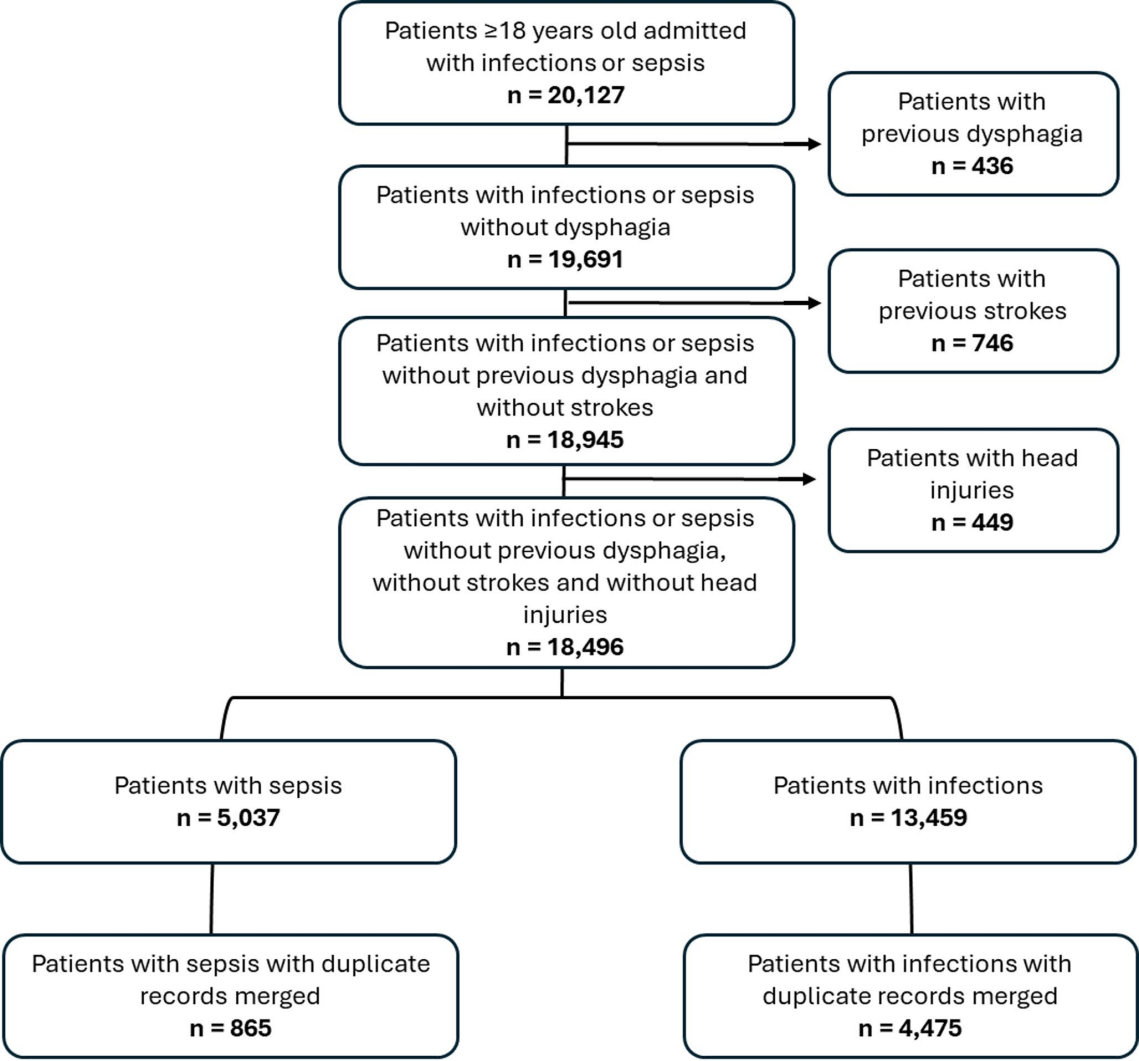
Study flow diagram

The initial screening of admission data revealed 18,496 entries of interest within the study period. This included 13,459 infections and 5,037 cases of sepsis (Fig. 1). After removal of duplicate data, and merging of information pertaining to the same inpatient stay, the total number of patients fell to 5340, of which 4475 (Pneumonia 2465, UTI 1888 and Cholecystitis 401) had infections, and 865 (Pneumonia 535, UTI 413 and Cholecystitis 55) had infections and sepsis (Table 1). Patients admitted with infections (Males 2031, Females 2444) had a mean age of 67 (SD:21) years, while those with sepsis (Males 434, Females 431) had a mean age of 72 (SD:16). There were no missing data that required exclusion from analysis.

**Table 1:**
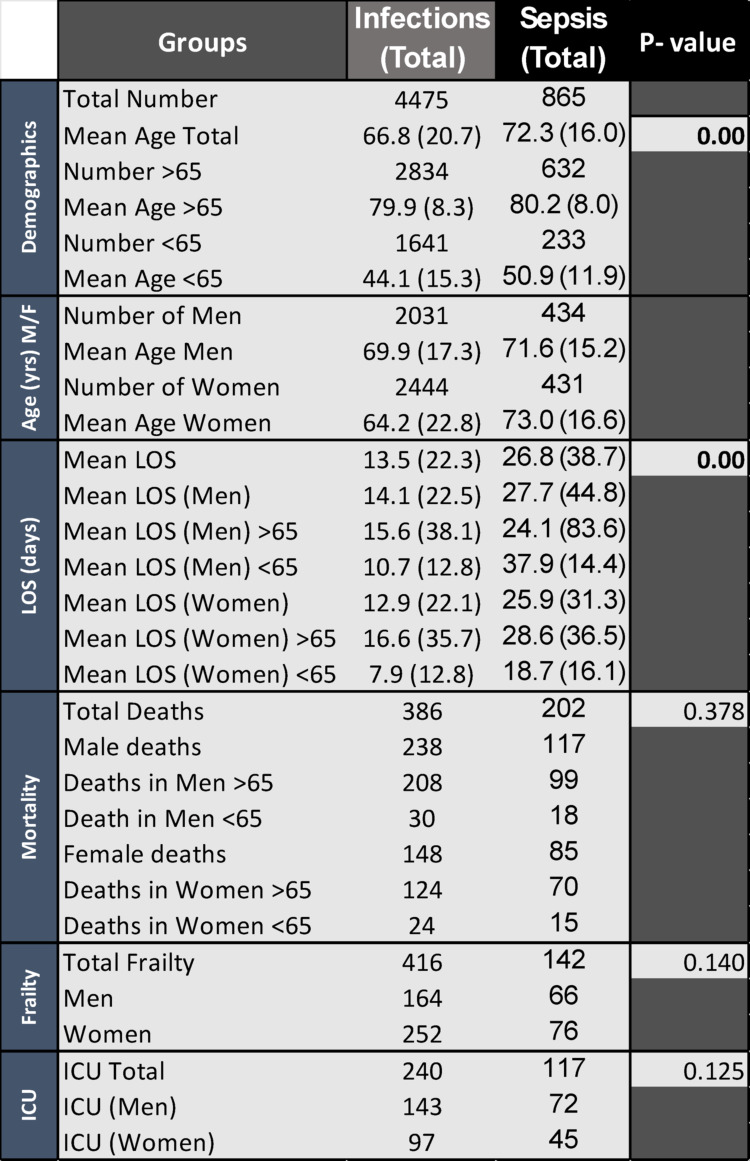
Total infection and sepsis data

### Infections and Dysphagia

In total, 189 (4.2%) patients with infections developed dysphagia during their inpatient stays (Table 2). Infection-associated dysphagia was seen in all types of infection studied. This included 148 (6.0%) patients with pneumonia, 67 (3.5%) patients with UTIs, and 3 (0.8%) patients with cholecystitis who developed dysphagia (Supplementary Table 1).

**Table 2:**
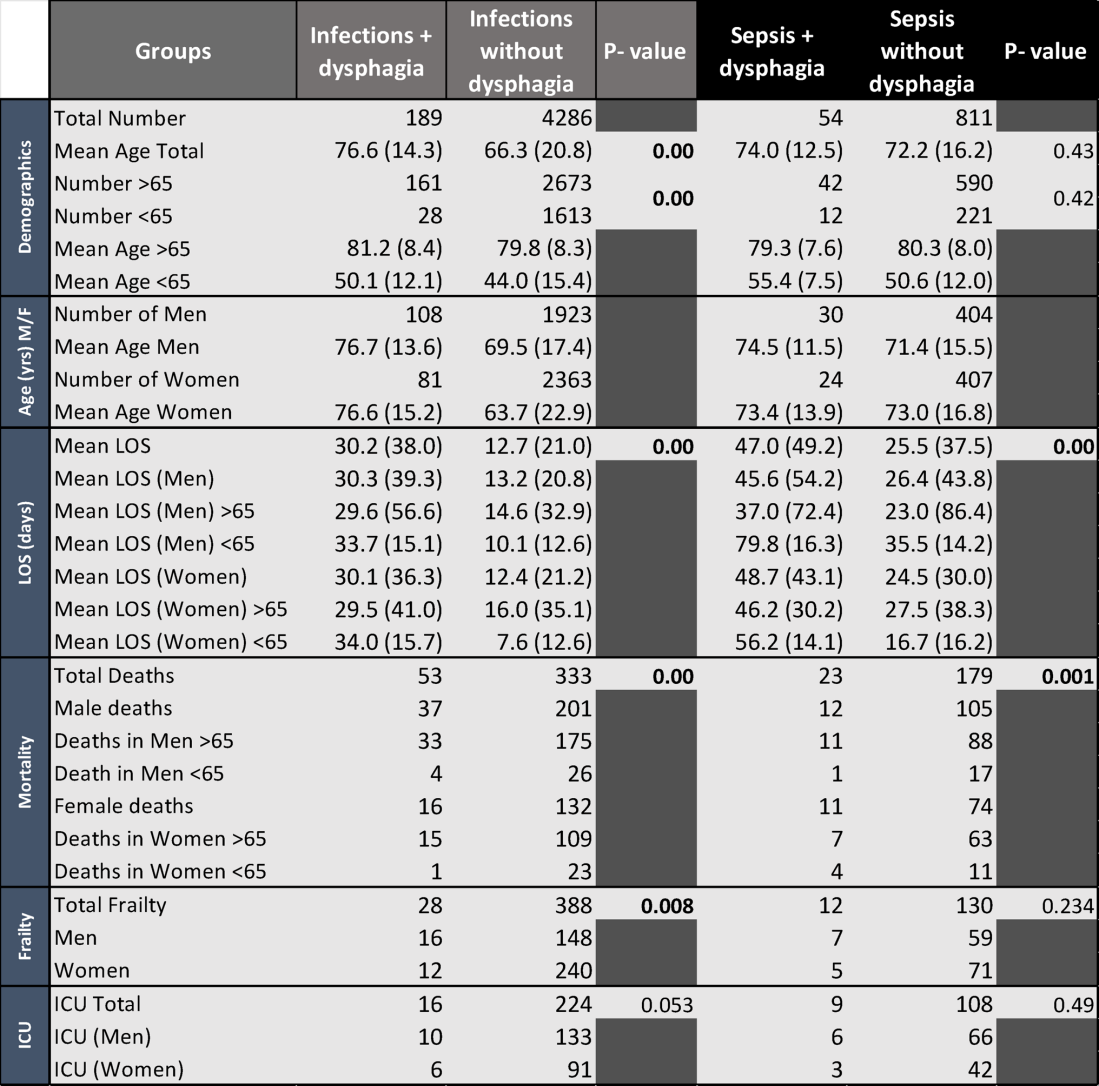
Infection and sepsis data for those with and without dysphagia

(Bold values are those that are statistically significant. Sections which are greyed out were not statistically compared using either T or Chi-Squared tests)

### Comparisons

Patients with dysphagia were significantly older (*P* < 0.0001), with a longer LOS (*P* < 0.0001), greater mortality (*P* < 0.0001) and were frailer (*P* < 0.0001) than those without dysphagia (Table 2). Further, comparisons between the characteristics and outcomes of patients with infections and dysphagia and those with infections but without dysphagia were made and can be seen in Table 2 and Supplementary Table 1.

### Co-morbidities and Complications

Concerning total infections, the most common co-morbidities affecting the largest number of patients were COPD 20.0% (898), CKD 19.5% (872) and obesity 19.3% (862). Delirium was also noted to affect a substantial percentage of patients, 18.6% (834). The distribution of co-morbidities and post-infective complications of interest for total and individual infections can be seen in Table 3 and Supplementary Table 3.

**Table 3:**
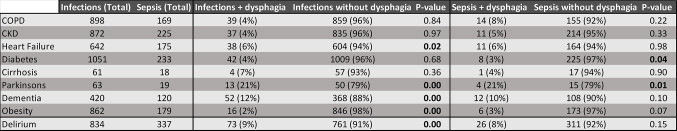
General comorbidity data

(Bold values are those that are statistically significant)

### Sepsis and Dysphagia

Fifty-four (6.2%) patients with sepsis developed dysphagia as inpatients (Table 2). Regarding individual types of sepsis, this included 50 (9.3%) patients with pneumonia, 14 (3.4%) patients with urosepsis and 2 (3.6%) patients with cholecystitis (Supplementary Table 2). As was the case for patients with infection-associated dysphagia, sepsis-associated dysphagia was observed to occur in all three types of sepsis studied.

### Comparisons

Patients with sepsis and dysphagia had significantly longer LOS (*P* < 0.0001) and greater mortality (P 0.001) compared to those without dysphagia. Additional comparisons between the characteristics of dysphagic and non-dysphagic patients for total sepsis and individual types of sepsis can be seen in Table 2 and Supplementary Table 2.

### Co-morbidities and Complications

The most commonly occurring comorbidities for patients admitted to hospital with sepsis were CKD 26.0% (225), HF 20.2% (175) and COPD 20.0% (169). As was seen in the data for patients with infections, a large proportion of patients, 39.0% (337), had delirium during their inpatient stays (Table 3 and Supplementary Table 4).

### Logistic Regression

Logistic regression was performed on the total data set to assess the relationships as well as the direction of effect between dysphagia and various variables of clinical interest. Several factors were found to increase the risk of dysphagia in patients admitted to hospital with infections. In decreasing order of effect size, these were the presence of PD, dementia, sepsis, delirium and age (OR 3.88, 2.43, 1.74, 1.60 and 1.02; *P* < 0.001, < 0.001, 0.013, 0.008 and 0.002). The presence of obesity and female gender were found to reduce the risk of dysphagia occurring (OR 0.53, 0.65; P 0.006, 0.001). No significant associations were observed for any other comorbidities (Table 4). ROC analysis revealed an AUC of 0.718, indicating acceptable discriminative ability. Furthermore, multicollinearity was assessed using tolerance and Variance Inflation Factor (VIF). All VIF values were below 2, and tolerance values above 0.2, indicating no significant multicollinearity (Supplementary Table 5).

**Table 4:** Logistic regression

						Wald Test		
Model		Estimate	Standard Error	Odds Ratio	z	Wald Statistic	df	*p*
M₁	(Intercept)	−4.180	0.352	0.015	−11.890	141.366	1	**< 0.001**
	Sepsis	0.552	0.223	1.737	2.479	6.146	1	**0.013**
	Dementia	0.889	0.193	2.433	4.611	21.258	1	**< 0.001**
	Delirium	0.470	0.177	1.600	2.655	7.052	1	**0.008**
	Parkinsons	1.356	0.336	3.879	4.032	16.257	1	**< 0.001**
	Gender	−0.438	0.137	0.645	−3.200	10.243	1	**0.001**
	Frailty	0.130	0.189	1.139	0.687	0.472	1	0.492
	COPD	0.065	0.165	1.067	0.394	0.155	1	0.693
	CKD	−0.313	0.174	0.731	−1.802	3.247	1	0.072
	HF	0.179	0.175	1.196	1.022	1.044	1	0.307
	Diabetes	−0.301	0.169	0.740	−1.787	3.192	1	0.074
	Cirrhosis	0.535	0.475	1.708	1.127	1.270	1	0.260
	Obesity	−0.641	0.232	0.527	−2.758	7.607	1	**0.006**
	Age	0.015	0.005	1.016	3.174	10.076	1	**0.002**

(Bold values are those that are statistically significant)

### Cox Regression

Several variables were associated with an increased LOS. In order of decreasing effect, these included dysphagia, sepsis, delirium, cirrhosis, frailty, HF and age (HR 0.59, 0.63, 0.67, 0.74, 0.79, 0.92, 0.99; *P* < 0.001, < 0.001, < 0.001, 0.009, < 0.001, 0.039 and < 0.001). Female patients had a slightly reduced LOS (HR 1.07; P 0.022) (Supplementary Table 6).

Concerning mortality, in decreasing order of effect, the variables which increased the risk of in hospital mortality were HF, male gender, sepsis, dementia, dysphagia, COPD, CKD and age (HR 1.67, 1.66, 1.55, 1.38, 1.37, 1.26, 1.22, 1.02; *P* < 0.001, < 0.001, < 0.001, 0.005, 0.012, 0.016, 0.036 and < 0.001). Unexpectedly, frailty reduced the risk of mortality (HR 0.65; *P* < 0.001) (Supplemental Table 7).

## Discussion

This study is the first to establish the prevalence of infection associated dysphagia in patients admitted to hospital without pre-existing dysphagia or strokes. Additionally, our study findings add to the slowly emerging literature pertaining to sepsis associated dysphagia. We observed that most infection or sepsis-associated dysphagia occurred in patients with no ICU exposure. This implies that, while the incidence of dysphagia is concentrated in the ICU setting, the greatest burden of sepsis and infection-associated dysphagia lies on medical and surgical wards affecting non critically unwell patients. Our findings also reveal that sepsis increases the risk of dysphagia to a degree comparable with well-known factors such as dementia and delirium. However, this retrospective study, by its nature, is unable to establish a causal link between infections, sepsis and dysphagia. Furthermore, the pathophysiological processes leading to the emergence of dysphagia in the context of infections and sepsis are yet to be established, leaving at least three plausible means by which dysphagia arises. These include by worsening an existing subclinical, asymptomatic, swallowing impairment, by unmasking an existing mild but unrecognised/undiagnosed dysphagia or by causing patients with otherwise normal swallowing physiology to decompensate. Despite more work needing to be done, both infection and sepsis-associated dysphagia should be borne in mind by clinicians when patients are admitted to hospitals.

The prevalence of infection and sepsis-associated dysphagia in our patient population were 4% and 6%, with sepsis-associated dysphagia occurring more frequently than infection-associated dysphagia. Though a substantial minority of patients admitted to hospital did have sepsis-associated dysphagia, our prevalence was lower than the reported prevalence in the three previous studies on this subject. The two studies performed in patients with severe sepsis in ICUs showed a dysphagia prevalence of 63% [8] and 57% [11], while the previous study in ward based patients with sepsis observed a prevalence of 16% [12]. Potential reasons for the large observed differences in prevalence include the relatively small number of patients in the previous studies, leading to potentially skewed findings, and the fact that critically unwell patients with severe sepsis on ICU are at risk of several specific dysphagia associated factors in addition to the dysphagia associated factors encountered in non critical care settings. These include CIP, critical illness and organ dysfunction, intubation, the presence of a tracheostomy, sedation and impaired consciousness [19–21]. Another reason is the fact that previous studies had patient populations that differed from the patient population in this study. The two ICU based studies focused on severe sepsis in critically unwell patients, while the Sasegbon et al. study [12], despite involving ward-based patients, only recruited patients ≥ 65 years of age. In this light, it is perhaps not entirely surprising that the prevalences we observed are dissimilar to the published literature, albeit more similar to the findings of the ward based study [12].

Dysphagia was also seen to occur for all types of infections and sepsis studied, albeit at differing rates. For both patients with sepsis and those with infections, dysphagia occurred most frequently with pneumonia. This pattern was also seen in all three previous studies in this field [8, 11, 12]. The reason for this association remains unclear, but several factors may be at work. One explanation is that as it is known that aspiration can lead to pneumonia, there is a risk that, within the studies, pneumonia may have developed as a consequence of dysphagia rather than being its cause. Because formal swallowing assessments do not routinely take place prior to or at the point of admission, it can be difficult to establish with absolute certainty that pneumonia preceded dysphagia. To mitigate this risk as much as possible, this study sought to exclude all patients with previous dysphagia to reduce the risk of underlying dysphagia causing aspiration and subsequent pneumonia. To further reduce this risk, this study focused on the diagnosis of infection or sepsis made by the admitting clinical team. Pneumonia and aspiration pneumonia have different codes, which means that when each diagnosis of pneumonia was made, there was no clinical suspicion that what was observed was aspiration pneumonia. Furthermore, it must be noted that dysphagia has been shown in this and previous studies to be associated with sepsis from other organ systems. This means that unrecognised aspiration cannot be viewed as the full picture.

Another explanation is that that some infective organisms which cause chest infections may also cause inflammation of the oropharynx, leading to altered swallowing behaviour and oropharyngeal functionality, thereby increasing the risk of dysphagia [22]. Aditionally, infective exacerbations of COPD have been shown to alter the natural breathing and swallowing cycle [23], thereby increasing the risk of dysphagia. Similar disruptions to the breathing-swallowing cycle in patients with pneumonia may explain the increased prevalence of sepsis and infection-associated dysphagia that we observed.

In the Sasegbon et al. study, it was hypothesised that sepsis-associated dysphagia occurred in older participants due to a combination of sepsis induced neurological impairment and catabolysis, resulting in relative oropharyngeal muscle wasting and subsequent weaker and discoordinated [12] muscle contractions. However, as to why infections without sepsis may increase the risk of dysphagia is unclear. It may be because infections in the absence of sepsis have also been shown to cause catabolysis, muscle breakdown and impaired functionality [24]. Although the study design does not allow for causal conclusions, the fact that we observed that patients with sepsis have a higher prevalence of dysphagia than those with infections but without sepsis may reflect demographic differences between the two groups, with patients with infections but without sepsis being found to be significantly younger than those with sepsis. Alternatively, this observation may be due to a comparatively lower degree of catabolysis and oropharyngeal muscle impairment in patients with infections compared to those with sepsis. Though plausible, this hypothesis is unsupported by any studies and, as such, should be treated with caution. It is our suspicion, albeit based on a limited amount of available evidence, that infection and sepsis associated dysphagia is part of a complex interplay of factors, including respiratory-swallowing discoordination, neuroinflammatory changes affecting central control of the swallowing sensorimotor system, and neuromuscular dysfunction. In this context, dysphagia arises by one or more processes whereby, as mentioned earlier, there is worsening of subclinical asymptomatic swallowing impairment, such as presbyphagia, the unveiling of previously unrecognised/undiagnosed mild dysphagia, or the decompensation of a patient with previously normal swallowing function.

No previous study on sepsis-associated dysphagia has attempted to associate as many factors with the occurrence of dysphagia. Previous studies have focused on important factors known to cause and be associated with dysphagia in ICU, such as endotracheal intubation and organ failure [8, 11], whereas in this study, we sought to study common comorbidities that patients possess at the point of admission. This was with the aim of helping to inform the decision making of clinicians when admitting patients with infections or sepsis to hospitals. Viewed as a whole, this study, along with previously published studies in the field, provides valuable information enhancing our understanding of the factors associated with sepsis associated dysphagia. Logistic regression revealed that PD, sepsis, delirium, dementia and increasing age were all significantly associated with an increased risk of dysphagia. PD, delirium, and dementia are well-known risk factors for dysphagia [25–28], and as such, it was perhaps unsurprising to see that they significantly and strongly increased the risk of dysphagia occurring. Interestingly, sepsis exerted the third strongest effect, after PD and dementia, at increasing the risk of dysphagia with a notably greater effect than delirium, which is well known to cause dysphagia. Notably, delirium, an acute and temporary impairment in cognition which often affects elderly patients and is frequently caused by sepsis [1, 29, 30], is a plausible alternative candidate which explains infection and sepsis associated dysphagia. Therefore, the occurrence and resolution of dysphagia in the context of sepsis may reflect the occurrence and resolution of delirium. Though interesting, this possible explanation does not fully explain what has been observed, as infection and sepsis associated dysphagia has been seen to occur in patients without delirium in this and previous studies. Furthermore, as our study design sought to exclude any patients with preexisting dysphagia from the study, this meant that despite the presence of these known risk factors, no patients with PD, delirium or dementia were known to have had dysphagia previously until a significant proportion of them decompensated on admission to hospital. In this light, clinicians should be aware of the increased risk of dysphagia for all patients with sepsis as well as for those with PD, dementia or delirium. Increasing patient age was also significantly associated with dysphagia, but weakly so. No other comorbidity assessed was found to be significantly associated with dysphagia. Obesity and female gender were found to reduce the risk of dysphagia occurring. The precise reasons for this are unclear. At present, there is no consensus as to the effects of obesity on muscle strength, notwithstanding the recognised condition of sarcopenic obesity [31]. Some studies suggest that patients who are obese have a reduced risk of sarcopenia [32], while others suggest that obesity causes muscle wasting [33]. Unfortunately, despite the ICD-10 code for obesity being used frequently in our dataset, the code for sarcopenia was not used. The reasons for this were unclear. Furthermore, as information pertaining to body mass index (BMI) during each patient’s admission could not be easily extracted from patient records, this rather interesting finding could not be further explored and requires further evaluation in future prospective studies.

Regarding Cox regression, we observed that dysphagia and sepsis were the factors with the strongest effect on prolonging inpatient hospital stays and significantly increased inpatient mortality, compared to other significant factors such as delirium, age, HF, etc. This tallied with the focus of this study and is in keeping with the literature, where studies have shown that the presence of any of these factors can prolong discharges and increase mortality [34–39]. Interestingly, female gender was noted to significantly reduce LOS and in-hospital mortality as well as reduce the risk of dysphagia. Although co-morbidities were not directly compared between genders, these findings, viewed together, are suggestive of the female patients in our study being less comorbid on admission than men, leading to a reduced risk of sepsis/infection-related complications. The observation that female patients tend to be less comorbid than their male counterparts has been observed in previous sepsis studies [40]. Unexpectedly, our data showed that frailty was associated with reduced mortality, despite being predictably linked to increased length of stay (LOS). This was a counterintuitive finding and warrants cautious interpretation. The reasons for this are unclear but may reflect an artefact of coding or a combination of selection bias and survivor effect in some frail patients, whereby those who are more frail are admitted to hospital earlier and receive more intensive monitoring and support [41].

Despite the real-world strengths of this study, it has several weaknesses which should be taken into account when considering its findings. Firstly, this study relies on clinical coding data from patients’ EPRs. Studies have shown that, despite attempts to improve practice and the best attempts of clinical and administrative teams, this data is not always perfectly accurate [15]. In this context, the statistical effect of inaccurate coding would be to increase variability and reduce the effect size of observed differences between groups. This would increase the type 2 error rate and result in an inappropriate acceptance of the null hypothesis [16]. This source of potential and unquantifiable inaccuracy means that study findings should be interpreted with appropriate caution. However, the fact that significant differences were observed despite this known issue is notable. It should also be noted that we chose to focus on three types of infection and sepsis in this study to ensure feasibility and clinical relevance. However, this approach excluded other types of infections and sepsis. Another limitation is the fact that this study was retrospective. Performing the study in this manner meant that while associations can be shown, causation cannot be proven. Furthermore, residual confounding remains a distinct possibility despite multivariable adjustment. To try to mitigate these limitations, the research team worked closely with the expert clinical coding and electronic patient record teams to try to ensure that the coding and clinical information were as accurate as possible. Due to the number of variables, the research team ensured that there were more than 10 EPV to avoid data distortion by overfitting. Furthermore, multicollinearity was assessed as part of the model evaluation process. However, despite our checks to limit model overfitting and multicollinearity, which resulted in the final model showing acceptable discrimination (AUC 0.718), unexplained variance remains, and the possibility of unmeasured confounding persists. Another limitation is the fact that dysphagia was diagnosed clinically based on ward suspicion and subsequent evaluation by speech and language therapists. While this is reflective of widespread clinical practice, there is a potential for subtle dysphagia to be overlooked by ward based clinical teams and for speech and language therapy referrals not to be made, ultimately resulting in missed cases of dysphagia. In addition, the absence of baseline instrumental swallowing assessments at the point of admission impairs our ability to delineate onset and recovery dynamics. Furthermore, although we excluded patients with known prior dysphagia, unrecognised pre-existing dysphagia cannot be completely excluded. This raises the possibility that the presence of infection or sepsis unmasked unknown dysphagia in some patients. Lastly, this study focused solely on in-hospital infection and sepsis-associated dysphagia and did not follow patients up post discharge. Hence, the recovery of dysphagia post discharge requires further exploration.

Taken together, these limitations highlight the need for future prospective, preferably multicentre, studies. Such work should include formal clinical and instrumental swallowing assessments at the point of admission, with repeated evaluations during the inpatient stay and beyond. Prospective studies will also allow for clearer case definitions better distinguishing oropharyngeal from oesophageal dysphagia, and separating aspiration pneumonia from other respiratory infections. Future studies should also capture detailed information on nutritional status, BMI and sarcopenia, delirium severity, medication exposures, and the timing and nature of rehabilitation interventions, alongside illness severity. Longer-term outcomes, including persistence or recovery of dysphagia and its impact on readmissions and mortality, should be assessed. These measures would allow a more accurate understanding of temporality, refine effect estimates, and clarify whether observed associations reflect causality or confounding. Ultimately, increased awareness of infection and sepsis-associated dysphagia should result in proactive swallowing assessments of patients with infections and sepsis as they are admitted to hospital and prompt, where appropriate, pre-emptive intervention in those deemed to be at particularly high risk.

In conclusion, we have shown that infections and sepsis are associated with dysphagia. Patients with infections and sepsis often have other factors known to be associated with and to cause dysphagia. Therefore, the emergence of dysphagia may reflect a worsening of existing subclinical swallowing impairment in the context of infection and sepsis-associated physiological stress. Older patients and those with PD, delirium or dementia without previous recognised dysphagia are at high risk of developing dysphagia or having their dysphagia unmasked should they be admitted with infections or sepsis.

## Supplementary Information

Below is the link to the electronic supplementary material.

Supplementary Material 1 (PDF 389 KB)

## Data Availability

The data that support the findings of this study are not openly available due to reasons of sensitivity and are available from the corresponding author upon reasonable request.
