# Supplementary material for: The Prevalence of Infection and Sepsis Associated Dysphagia in Hospitalised Patients: A Retrospective Cross-sectional Study
Source: Dysphagia. 2026 Mar 4;41(4):799–810. doi: 10.1007/s00455-026-10931-w (PMC13457454; doi:10.1007/s00455-026-10931-w)
Supplement: Supplementary file 1 [file 455_2026_10931_MOESM1_ESM.pdf]

Supplementary Table 1:

	Infections (without sepsis)									
	Groups	Pneumonia + dysphagia	Pneumonia without dysphagia	P-value	UTI + dysphagia	UTI without dysphagia	P-value	Cholecystitis + dysphagia	Cholecystitis without dysphagia	P-value
Demographics	Total Number	148	2317		67	1821		3	398	
	Mean Age Total	77.6 (13.1)	71.1 (18.4)	**0.00**	74.3 (15.3)	63.2 (22.8)	**0.00**	69.0 (27.8)	60.0 (17.9)	
	Number >65	128	1686	**0.00**	55	1036	**0.00**	2	166	
	Number <65	20	631		12	785		1	232	
	Mean Age >65	81.4 (8.7)	80.1 (8.4)		80.2 (8.1)	80.3 (8.0)		85.0 (2.8)	77.3 (7.9)	
	Mean Age <65	53.7 (11.2)	47.0 (16.0)		47.3 (10.8)	40.8 (15.2)		37.0 (-)	47.6 (11.7)	
Age (yrs) M/F	Number of Men	88	1180		31	674		3	181	
	Mean Age Men	76.7 (13.1)	70.2 (17.8)		75.5 (14.3)	71.4 (16.2)		69.0 (27.8)	63.6 (16.2)	
	Number of Women	60	1137		36	1147		0	217	
	Mean Age Women	79.0 (13.1)	72.0 (18.9)		73.3 (16.2)	58.5 (24.7)		(-)	57.0 (18.8)	
LOS (days)	Mean LOS	30.6 (40.5)	14.9 (21.9)	**0.00**	48.2 (51.9)	14.1 (24.3)	**0.00**	17.0 (17.6)	6.0 (11.7)	
	Mean LOS (Men)	30.6 (41.5)	14.6 (21.5)		52.6 (58.1)	15.1 (24.4)		17.0 (17.6)	7.9 (14.3)	
	Mean LOS (Men) >65	29.7 (61.2)	15.6 (34.0)		53.7 (72.6)	16.8 (28.8)		23.5 (-)	11.4 (18.9)	
	Mean LOS (Men) <65	35.3 (15.0)	12.1 (12.6)		47.0 (18.6)	10.4 (14.2)		4.0 (17.6)	4.9 (9.0)	
	Mean LOS (Women)	30.5 (39.3)	15.1 (22.3)		44.4 (46.3)	13.6 (24.3)		(-)	4.4 (8.8)	
	Mean LOS (Women) >65	29.6 (44.0)	16.0 (35.6)		46.6 (42.6)	21.1 (34.7)		(-)	6.5 (12.3)	
	Mean LOS (Women) <65	38.3 (16.1)	12.8 (13.1)		35.3 (14.4)	6.8 (13.6)		(-)	3.1 (7.2)	
Mortality	Total Deaths	48	302	**0.00**	13	51	**0.00**	1	8	
	Male deaths	33	186		9	27		1	5	
	Deaths in Men >65	29	162		8	27		1	3	
	Death in Men <65	4	24		1	0		0	2	
	Female deaths	15	116		4	24		0	3	
	Deaths in Women >65	14	99		3	18		0	2	
	Deaths in Women <65	1	17		1	6		0	1	
Frailty	Total Frailty	23	280	0.214	10	141	**0.033**	0	9	
	Men	15	112		4	47		0	5	
	Women	8	168		6	94		0	4	
ICU	ICU Total	12	166	0.667	9	69	**0.00**	0	10	
	ICU (Men)	8	104		5	34		0	7	
	ICU (Women)	4	62		4	35		0	3	

(Bold values are those that are statistically significant. Sections which are greyed out were not statistically compared using either T or Chi-Squared tests)

Supplementary Table 2:

	Sepsis									
	Groups	Pneumonia + dysphagia	Pneumonia without dysphagia	P-value	UTI + dysphagia	UTI without dysphagia	P-value	Cholecystitis + dysphagia	Cholecystitis without dysphagia	P-value
Demographics	Total Number	50	485		14	399		2	53	
	Mean Age Total	74.1 (12.6)	73.5 (15.7)	0.782	74.4 (9.9)	72.2 (16.2)	0.623	82.0 (4.2)	70.0 (15.9)	
	Number >65	39	371	0.81	12	286	0.25	2	37	
	Number <65	11	114		2	113		0	16	
	Mean Age >65	79.5 (7.4)	80.5 (8.3)		77.1 (7.7)	80.6 (7.8)		82.0 (4.2)	78.6 (8.2)	
	Mean Age <65	55.2 (7.8)	50.6 (11.9)		58.0 (0)	51.0 (12.1)		(-)	50.0 (10.1)	
Age (yrs) M/F	Number of Men	28	251		8	185		2	29	
	Mean Age Men	74.4 (11.6)	71.4 (16.2)		73.6 (8.6)	73.7 (14.2)		82.0 (4.2)	66.1 (15.7)	
	Number of Women	22	234		6	214		0	24	
	Mean Age Women	73.7 (14.0)	75.8 (14.9)		75.3 (12.3)	70.9 (17.8)		(-)	74.6 (15.1)	
LOS (days)	Mean LOS	46.9 (48.8)	30.2 (44.0)	**0.012**	77.2 (76.4)	28.0 (44.6)	**0.00**	42.5 (50.2)	27.4 (34.6)	
	Mean LOS (Men)	48.1 (55.3)	32.23 (51.8)		73.6 (90.3)	28.2 (54.4)		42.5 (50.2)	31.1 (41.8)	
	Mean LOS (Men) >65	39.5 (72.4)	27.9 (91.2)		44.1 (106.6)	24.1 (111.6)		42.5 (-)	17.8 (61.7)	
	Mean LOS (Men) <65	79.8 (16.5)	44.0 (15.0)		280.0 (16.1)	42.6 (13.4)		(-)	50.1 (14.4)	
	Mean LOS (Women)	45.4 (40.3)	28.0 (33.6)		82.0 (60.7)	27.7 (34.0)		(-)	23.0 (23.3)	
	Mean LOS (Women) >65	47.1 (29.4)	30.0 (40.3)		70.6 (2.9)	32.6 (41.5)		(-)	26.0 (-)	
	Mean LOS (Women) <65	39.6 (14.4)	19.8 (16.6)		139.0 (4.5)	18.1 (17.3)		(-)	7.8 (12.6)	
Mortality	Total Deaths	23	139	**0.011**	4	71	0.304	0	5	
	Male deaths	12	82		2	40		0	4	
	Deaths in Men >65	11	69		2	37		0	3	
	Death in Men <65	1	13		0	3		0	1	
	Female deaths	11	57		2	31		0	1	
	Deaths in Women >65	7	48		2	27		0	1	
	Deaths in Women <65	4	9		0	4		0	0	
Frailty	Total Frailty	11	81	0.344	3	66	0.63	0	7	
	Men	7	36		1	31		0	3	
	Women	4	45		2	35		0	4	
ICU	ICU Total	9	76	0.668	3	45	0.244	0	7	
	ICU (Men)	6	52		2	22		0	6	
	ICU (Women)	3	24		1	23		0	1	

(Bold values are those that are statistically significant. Sections which are greyed out were not statistically compared using either T or Chi-Squared tests)

Supplemental Table 3:

	Infections								
	Pneumonia + dysphagia	Pneumonia without dysphagia	P-value	UTI + dysphagia	UTI without dysphagia	P-value	Cholecystitis + dysphagia	Cholecystitis without dysphagia	P-value
COPD	31	696	**0.02**	12	213	0.12	1	14	
CKD	30	477	0.93	11	404	0.26	0	25	
Heart Failure	29	485	0.70	15	169	**0.00**	1	12	
Diabetes	35	599	0.55	19	409	0.26	0	80	
Cirrhosis	3	33	0.55	2	18	0.12	0	8	
Parkinsons	11	31	**0.00**	3	24	**0.03**	0	0	
Dementia	42	230	**0.00**	15	170	**0.00**	0	149	
Obesity	11	419	**0.00**	6	313	0.09	0	1	
Delirium	58	492	**0.00**	30	355	**0.00**	0	0	

(Bold values are those that are statistically significant. Sections which are greyed out were not statistically compared using the Chi-Squared test)

Supplemental Table 4:

	Sepsis								
	**Pneumonia + dysphagia**	**Pneumonia without dysphagia**	**P-value**	**UTI + dysphagia**	**UTI without dysphagia**	**P-value**	**Cholecystitis + dysphagia**	**Cholecystitis without dysphagia**	**P-value**
COPD	13	122	0.90	3	62	0.55	1	3	
CKD	11	122	0.62	3	119	0.50	0	16	
Heart Failure	10	122	0.42	1	63	0.38	1	5	
Diabetes	7	127	0.06	3	125	0.43	1	11	
Cirrhosis	1	11	0.90	0	6	0.64	0	1	
Parkinsons	4	8	**0.00**	2	9	**0.01**	0	0	
Dementia	12	61	0.29	0	66	0.80	0	13	
Obesity	5	106	**0.03**	1	87	0.10	0	1	
Delirium	24	200	0.36	6	162	0.87	0	0	

(Bold values are those that are statistically significant. Sections which are greyed out were not statistically compared using the Chi-Squared test)

Supplementary Table 5:

Multicollinearity Diagnostics

	Tolerance	VIF
Sepsis	0.523	1.911
Dementia	0.653	1.531
Delirium	0.600	1.667
Parkinsons	0.741	1.349
Gender	0.969	1.032
Frailty	0.933	1.072
COPD	0.961	1.041
Obesity	0.960	1.042
Age	0.736	1.359
CKD	0.926	1.079
HF	0.921	1.085
Diabetes	0.950	1.052
Cirrhosis	0.988	1.012

Supplementary Table 6:

Cox Regression (LOS)

Cox Proportional Hazards Estimates Table

Model		Estimate	Standard Error	95% CI Lower	95% CI Upper	z	p
H_1	Age	-0.010	8.555×10^{-4}	-0.012	-0.009	-12.001	< .001
	Gender	0.064	0.028	0.009	0.118	2.292	0.022
	Frailty	-0.240	0.046	-0.331	-0.149	-5.184	< .001
	COPD	-0.027	0.035	-0.096	0.043	-0.760	0.447
	CKD	-0.014	0.035	-0.083	0.056	-0.389	0.697
	HF	-0.082	0.040	-0.160	-0.004	-2.063	0.039
	Diabetes	-0.045	0.034	-0.111	0.021	-1.343	0.179
	Cirrhosis	-0.298	0.114	-0.521	-0.075	-2.617	0.009
	Parkinsons	-0.143	0.112	-0.363	0.077	-1.275	0.202
	Obesity	0.040	0.036	-0.030	0.109	1.111	0.267
	Dysphagia	-0.522	0.066	-0.652	-0.392	-7.855	< .001
	Sepsis	-0.465	0.038	-0.539	-0.391	-12.291	< .001
	Delirium	-0.397	0.037	-0.469	-0.325	-10.805	< .001
	Dementia	0.034	0.049	-0.062	0.131	0.694	0.488

Hazard Ratios Estimates Table

Model		Hazard Ratio	95% CI Lower	95% CI Upper
H_1	Age	0.990	0.988	0.991
	Gender	1.066	1.009	1.126
	Frailty	0.786	0.718	0.861
	COPD	0.973	0.908	1.043
	CKD	0.986	0.920	1.057
	HF	0.921	0.852	0.996
	Diabetes	0.956	0.895	1.021
	Cirrhosis	0.743	0.594	0.928
	Parkinsons	0.867	0.695	1.080
	Obesity	1.040	0.970	1.116
	Dysphagia	0.593	0.521	0.676
	Sepsis	0.628	0.583	0.677
	Delirium	0.672	0.625	0.722
	Dementia	1.035	0.939	1.140

Supplemental Table 7:

Cox Regression (Mortality)

Cox Proportional Hazards Estimates Table

Model		Estimate	Standard Error	95% CI		z	P
				Lower	Upper		
H_1	Age	0.017	0.004	0.010	0.024	4.821	< .001
	Frailty	-0.427	0.124	-0.671	-0.183	-3.430	< .001
	COPD	0.228	0.095	0.043	0.413	2.413	0.016
	CKD	0.197	0.094	0.013	0.381	2.100	0.036
	HF	0.513	0.094	0.330	0.696	5.485	< .001
	Diabetes	-0.162	0.098	-0.354	0.030	-1.654	0.098
	Cirrhosis	0.284	0.255	-0.217	0.784	1.110	0.267
	Parkinsons	-0.025	0.257	-0.528	0.479	-0.095	0.924
	Obesity	-0.110	0.121	-0.347	0.127	-0.913	0.361
	Dysphagia	0.316	0.125	0.070	0.562	2.519	0.012
	Sepsis	0.437	0.089	0.264	0.611	4.936	< .001
	delirium	-0.047	0.093	-0.228	0.135	-0.507	0.612
	dementia	0.318	0.113	0.096	0.541	2.808	0.005
	Gender (M)	0.507	0.086	0.339	0.675	5.906	< .001

Hazard Ratios Estimates Table

Model		Hazard Ratio	95% CI	
			Lower	Upper
H_1	Age	1.017	1.010	1.024
	Frailty	0.652	0.511	0.833
	COPD	1.256	1.044	1.512
	CKD	1.218	1.013	1.464
	HF	1.670	1.391	2.006
	Diabetes	0.850	0.702	1.030
	Cirrhosis	1.328	0.805	2.191
	Parkinsons	0.976	0.590	1.614
	Obesity	0.895	0.707	1.135
	Dysphagia	1.372	1.073	1.754
	Sepsis	1.548	1.302	1.842
	Delirium	0.954	0.796	1.144
	Dementia	1.375	1.101	1.717
	Gender (M)	1.660	1.403	1.965
